# SARS‐CoV‐2 targeting by RNAi and host complement inhibition: A two‐pronged subterfuge for COVID‐19 treatment

**DOI:** 10.1002/iid3.549

**Published:** 2021-10-13

**Authors:** Raza Ali Naqvi, Deepak Shukla, Afsar R. Naqvi

**Affiliations:** ^1^ Department of Periodontics, College of Dentistry University of Illinois at Chicago Chicago Illinois USA; ^2^ Department of Ophthalmology and Visual Sciences University of Illinois Medical Center Chicago Illinois USA; ^3^ Department of Microbiology and Immunology University of Illinois Medical Center Chicago Illinois USA

**Keywords:** complement, gene regulation processes, infections, molecules, viral/retroviral

## Abstract

**Background:**

The lack of knowledge about the specific preventive measures and limited scientific information on severe acute respiratory syndrome coronavirus 2 (SARS‐CoV‐2) led to an excruciating onset and progression of coronavirus disease 2019 (COVID‐19). Swift development of various successful vaccines around the globe is striving to contain the exponential surges of COVID‐19 cases. However, the ongoing struggle to vaccinate the global population and alarming spread of highly transmissible variants may thwart global initiatives to contain SARS‐CoV‐2 as observed by less robust protective immunity.

**Methods:**

In this perspective, we propose a thought‐provoking, two‐pronged strategy involving RNA interference approach to degrade essential SARS‐CoV‐2 ORFs required for replication and entry in conjunction with a complement inhibitor (compstatin) to stymie the detrimental proinflammatory cytokine storm that exacerbate disease progression and severity.

**Results:**

We provide supporting evidence suggesting that concurrent targeting of viral and host components will be a superior strategy to effectively suppress viral spread and clinical manifestations of COVID‐19.

**Conclusion:**

SARS‐CoV‐2 specific RNAi in conjunction with systemic delivery of compstatin will be an effective two‐pronged strategy to combat local and systemic immune responses in both symptomatic and asymptomatic COVID‐19 patients.

## Graphical abstract

(1). Multiplex RNA interference (RNAi) can target SARS‐CoV‐2 essential transcripts and its emerging variants. (2). Complement C3a inhibitor compstatin can curtail the cytokine storm in COVID‐19 patients. (3). A novel RNAi and compstatin approach will contain virus and mitigate its downstream perils.

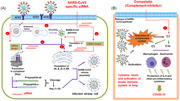

Severe acute respiratory syndrome coronavirus 2 (SARS‐CoV‐2) emerged in 2019 as one of the highly contagious viruses that has recently endangered the global human health due to life‐threatening “coronavirus disease 2019” (COVID‐19).^1^ Though it was initially identified as pneumonia outbreak on 31 December, 2019 from Wuhan Municipal Health Commission,^2^ the isolation of virus from bronchoalveolar lavage fluid samples from these patients and metagenomic RNA sequencing by the independent groups of researchers unraveled it as betacoronavirus that had never been reported so far in the human history.^3^, ^4^ In this pursuit, on 30 January, 2021 World Health Organization asserted it as a novel coronavirus outbreak and thereafter, International Committee on Taxonomy of Viruses' named this novel virus as “SARS‐CoV‐2”.^5^ Symptomatic COVID‐19 patients predominantly exhibit fever and cough, however, following warning signs and symptoms can also be present: sore throat, shortness of breath, fatigue, anosmia, dysgeusia.^3^, ^6^ In some patients extrapulmonary manifestations are also reported.^7^

The SARS‐CoV‐2 genome is a positive‐sense, single‐stranded, ribonucleic acid (RNA) of ~30 kb length. Spike (S) protein of SARS‐CoV‐2 plays important roles in the progression of COVID‐19. Ultradense peptide microarray confirmed that illness severity in COVID‐19 patients significantly increases the reactivity to nine different SARS‐CoV‐2 epitopes mapping key virulent factors including S, M, N, and ORF3a.^8^ Furthermore, antibodies against N‐terminal domain (NTD) and receptor binding domains (RBD) located in S1 and S2 subunits of S protein are proven as neutralizing antibodies and driven to inhibit SARS‐CoV‐2 infection via interfering with viral entry and membrane fusion.^9^ To date, multiple emergency use vaccines (against S‐proteins) are authorized based on their large phase III field efficacy clinical trials.^10^ Vaccines have proven highly successful preventative measure against SARS‐CoV‐2, thus far. However, challenges regarding the durable antiviral immunity in vaccinated population and efficient protection from the recently identified SARS‐CoV‐2 variants (specifically delta variant) have emerged and impeded the global response to contain pandemic. Recent data monitoring by CDC suggest vaccines are less effective against confirmed variant (beta, gamma, and delta) infection and symptomatic disease. It is therefore imperative to develop novel therapeutic strategies and treatment modalities to target SARS‐CoV‐2 genome and its subsequent clinical manifestations in addition to vaccine based preventive measures for COVID‐19 threat. In this perspective, we propose a combinatorial approach to repress multiple viral transcripts by RNA interference (RNAi) and dampen the host immune response to prevent COVID‐19 morbidity/mortality.

Multiple clinical trials and translational studies have uncovered the enormous therapeutic potential of RNAi that can be easily harnessed for targeted development of novel drugs. Posttranscriptional targeting of pathological transcripts and subsequent protein reduction by small interfering RNAs (siRNAs) is the crux of this technology. RNAi has been successfully used against various viruses viz., Hepatitis C Virus,^11^ poliovirus,^12^ etc. Thus, it can be used as a plausible stratagem to control SARS‐CoV‐2 load and eventually the downstream nefarious clinical manifestations of COVID‐19. This approach can be applied by introducing synthetic siRNAs (19–27 nucleotide long double‐stranded RNAs), or plasmid based in‐situ production of short hairpin RNAs.

Vir Biotechnology and Alnylam Pharmaceuticals strived jointly to uncover the possibility of treating COVID‐19 via RNAi (Alnylam Pharmaceuticals, 2020). A total 350 siRNAs targeting all available SARS‐CoV and SARS‐CoV‐2 genomes has been synthesized by Alnylam Pharmaceuticals (Source: https://investors.alnylam.com/press-release?id=24796.). Previous studies have demonstrated in vivo delivery of encapsulated siRNA to lungs (∼35%) and other organs using intranasal or intravenous routes suggesting promising therapeutic value of siRNAs.^13^, ^14^ In this pursuit, Idris et al. has diligently showed an effective siRNA against SARS‐CoV‐2 infection and administered it in vivo using a novel lipid nanoparticle delivery system.^13^ Authors screened and identified three candidate siRNAs that can efficiently inhibit (≥90%) the virus in lungs either alone or in combination with one another. This study uncovers the hidden potential of RNAi approach to contain SARS‐CoV‐2.^15^ However, to overwhelm the problem associated with upcoming SARS‐CoV‐2 variant siRNA strategy should be upgraded and should be focused on multiple SARS‐CoV‐2 derived important vaccine targets (Figure 1). In our opinion, application of a targeted RNAi approach can ameliorate viral load in the lungs of COVID‐19 subjects.

**Figure 1:**
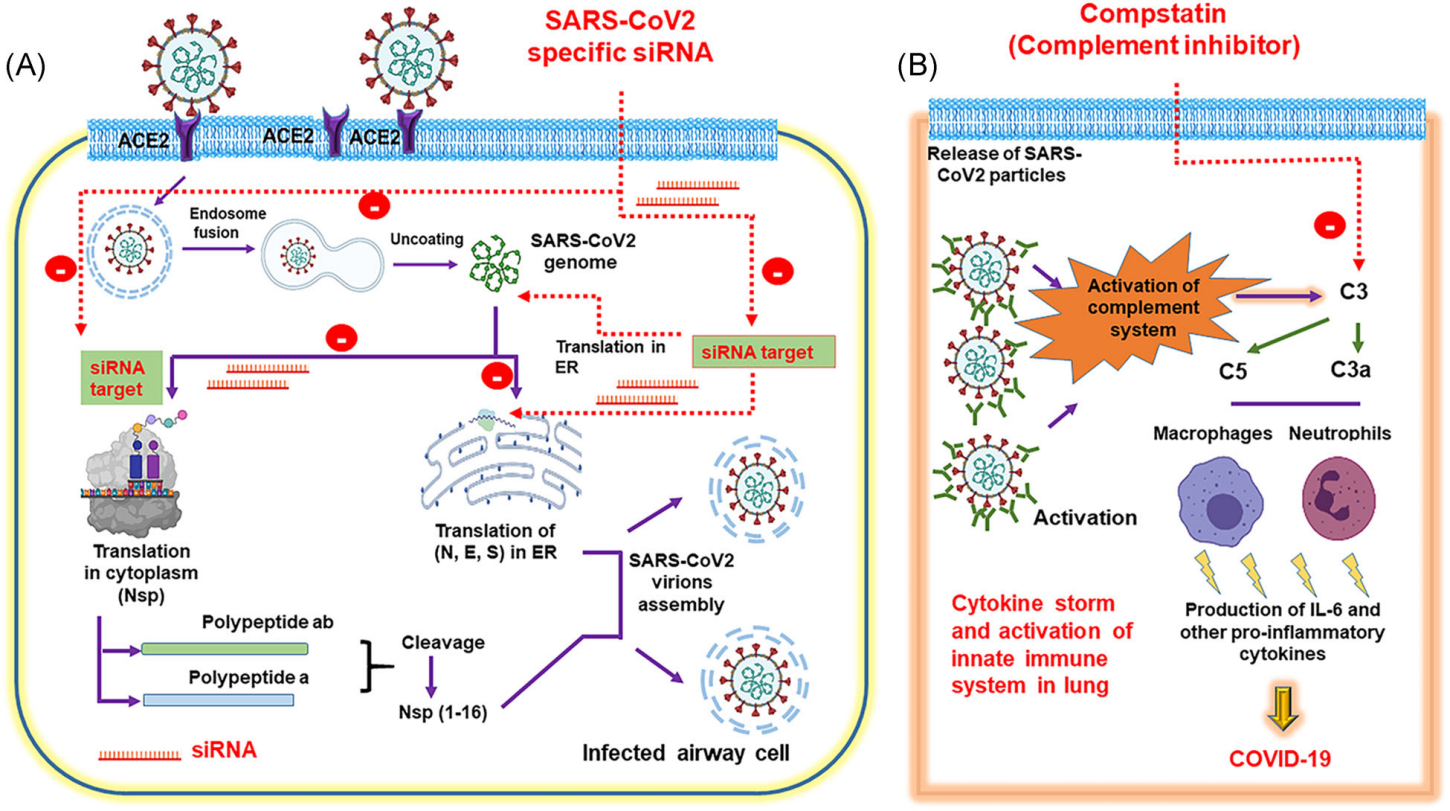
Two‐prong inhibitory strategy to suppress SARS‐CoV‐2 replication and clinical manifestation of COVID‐19. (A) Delivery of siRNA cocktail targeting multiple genes of SARS‐CoV‐2 to inhibit further generation of viral particles, and (B) Intravenous administration of complement inhibitor to alleviate the generation of IL‐6 and other proinflammatory cytokines, thereby attenuating the induction of cytokine storm: an attribute to COVID‐19. COVID‐19, coronavirus disease 2019; E, envelope protein; ER, endoplasmic reticulum; IL‐6, interleukin 6; N, nucleocapsid; Nsp, non structural protein; S, spike protein; SARS‐CoV‐2, severe acute respiratory syndrome coronavirus 2; siRNA, small interfering RNA

COVID‐19 is dictated by viral replication and the inflammatory host responses downstream to infection, leading to overt immune responses via activation of lung‐resident immune cells. Extravasation of neutrophils and monocytes into the bronchi cause disruption of the air–blood barrier by collateral damage of endothelial cells derived from airway cells and vascular endothelial cells expressing the ACE2 entry receptor for SARS‐CoV‐2. The damage of endothelial cells in this pursuit leads to thrombotic microangiopathies and the activation of complement system. Proinflammatory pathways triggered by complement are critical for antiviral innate immunity.^14^ Importantly, the strong staining of complement components mannose‐binding lectin (MBL), C4, C3, C5b‐9, in alveolar epithelial cells, inflammatory cells, pneumocytes, and exudates in alveolar spaces in the patients who died from COVID‐19 accentuated the role of complement system during pathogenesis of COVID‐19. Importantly, high throughout proteomics analysis demonstrated the upregulation of complement system proteins viz., C5, C6, and C8 the coagulation system, inflammation modulators, and proinflammatory factors in COVID19 patients.^15^, ^16^

Studying the role of complement activation in MERS‐CoV in humanized dipeptidyl‐peptidase 4 transgenic (hDPP4‐Tg) mice, Jiang et al. showed that MERS‐infected hDPP4‐Tg mice treated with anti‐C5a receptor (C5aR) antibody harbored significantly lower lung MERS‐CoV titers,^17^ less weight loss, reduced lung and spleen damage, lesser interferon‐gamma. These data suggest that inhibition of mice complement system can limit viral replication, suppress host immune response and may alleviate injury.^17^ Therefore, inhibiting complement activation/signaling after SARS‐CoV2 infection might be an effective immune therapeutic strategy to avoid the dire consequences of COVID‐19 (Figure 1). All complement pathways converge at C3 activation, inhibition of this component is the most reasonable way to attenuate the deleterious effects of complement system during the COVID‐19 pathogenesis. Interestingly, the derivatives of compstatin family (e.g., AMY‐101, Amyndas Pharmaceuticals) inhibits C3 and now being considered as a putative therapeutic agents in multiple clinical conditions.^18^ Interestingly, complement inhibitor compstatin is shown to interfere with interleukin‐6 (IL‐6) release ex vivo in a whole blood infection model.^19^ Gralinski et al.,^20^ showed that as compared to control C57BL/6J mice, SARS‐CoV‐infected *C3* ^–/–^ mice had significantly less weight loss and reduced respiratory dysfunction nevertheless comparable viral loads in the lung. Reduced infiltration of neutrophils and inflammatory monocytes was observed in the lungs of *C3* ^–/–^ mice as compared to the C56BL/6J controls.^21^ In addition, lower proinflammatory cytokine levels were detected in the sera of *C3* ^–/–^ mice.^21^ Together, complement system are critical in triggering a robust proinflammatory response during the pathogenesis of SARS‐CoV‐2 and its other family members. Hence, compstatin in COVID‐19 patients is expected to inhibit the severity of lung air‐blood barrier in bronchi by blocking C3a and the release of IL‐6 during activation of lung resident immune cells (Figure 1). In initial asymptomatic COVID‐19 patients, compstatin can further alleviate the extent of multiple organ failure by interfering with the activation of lung‐resident immune cells.

In conclusion, pulmonary injections of SARS‐CoV‐2 specific RNAi vectors (or inhalable modified siRNA/microRNA) and systemic delivery of compstatin will be an effective two‐prong strategy to combat local and systemic immune responses in both symptomatic and asymptomatic COVID‐19 patients. In our opinion, due to the specificity both in terms of SARS‐CoV‐2 replication/entry and curbing downstream cytokine storm, this regimen will surely have an edge over other on‐going trials (including various antiviral drugs, anti‐inflammatory agents and the antimalarial drug hydroxychloroquine) to treat COVID‐19 patients.

## CONFLICT OF INTERESTS

The authors declare that there are no conflict of interests.

## AUTHOR CONTRIBUTIONS

**Raza Ali Naqvi, Deepak Shukla, and Afsar R. Naqvi**: conceptualized and wrote the manuscript.
